# Re-wiring Guilt: How Advancing Neuroscience Encourages Strategic Interventions Over Retributive Justice

**DOI:** 10.3389/fpsyg.2020.00390

**Published:** 2020-03-13

**Authors:** Nathaniel E. Anderson, Kent A. Kiehl

**Affiliations:** ^1^The Mind Research Network, Albuquerque, NM, United States; ^2^Departments of Psychology, Neuroscience, and Law, University of New Mexico, Albuquerque, NM, United States

**Keywords:** neurolaw, neuroscience, jurisprudence, free will, determinism, culpability, intervention

## Abstract

The increasing visibility of neuroscience employed in legal contexts has rightfully prompted critical discourse regarding the boundaries of its utility. High profile debates include some extreme positions that either undermine the relevance of neuroscience or overstate its role in determining legal responsibility. Here we adopt a conciliatory attitude, reaffirming the current value of neuroscience in jurisprudence and addressing its role in shifting normative attitudes about culpability. Adopting a balanced perspective about the interaction between two dynamic fields (science and law) allows for more fruitful consideration of practical changes likely to improve the way we engage in legal decision-making. Neuroscience provides a useful platform for addressing nuanced and multifaceted deterministic factors promoting antisocial behavior. Ultimately, we suggest that shifting normative attitudes about culpability vis-à-vis advancing neuroscience are not likely to promote major changes in the way we assign legal responsibility. Rather, it helps us to shed our harshest retributivist instincts in favor of more pragmatic strategies for combating the most conspicuous patterns promoting mass incarceration and recidivism.

## Introduction

Increasing attention is being devoted to the emerging roles of neuroscience in legal decision-making, both in academic settings and in the courtroom. Among these roles is the growing influence neuroscience has in reinforcing more deterministic models of human decision-making and behavior. In a deliberate attempt to (over)simplify this complex landscape, it seems that there are roughly two camps in these conversations. The first includes those who promote the idea that neuroscience has mostly “disproved” the existence of free will, which subverts some of our ordinary notions of accountability. Prominent voices in popular media have indeed heralded the *end of free will* (Harris, 2012; Cave, 2016), calling into question our most basic presumptions about the legitimacy of punitive justice (Burns and Bechara, 2007; Sapolsky, 2017). As a consequence, the criminal justice system, which punishes people on a now-baseless presumption of freedom and agency, has been foundationally undermined and must therefore be replaced immediately with something more enlightened and fair. Expectedly, such claims have incited substantial opposition and motivated counter-arguments aimed at substantiating traditional views of legal responsibility and the enduring value of retributive sanctions against criminal actions. This opposing camp includes those who claim that free will (whether it exists or not) is largely irrelevant to basic notions of legal responsibility, and neuroscience has little to no relevance for assessing guilt or any ordinary sense of civic accountability. As such, the *status quo* can be safely perpetuated, and the law, as it stands, remains unfettered by trifling nuisances of pre-determined actions. Perhaps as a kind of contrecoup effect, these counter arguments often involve rebuffing the very relevance of neuroscience in the legal process more generally (Morse, 2006; Pardo and Patterson, 2010; Chambon and Bigenwald, 2019). Of course these descriptions are composite caricatures of many subtler perspectives available in this growing academic conversation, e.g. Vincent (2013). Still, many of the arguments we read these days overlap substantially and obviously with one of these two extreme positions. Without depreciating the zeitgeist of this revolution or its opponents, we recognize the need for some conservatism in advancing pragmatic attitudes about how the legal system might change in the wake of advancing neuroscience. We therefore set out here to willfully explore the vast middle ground between seemingly extreme perspectives in this conversation. We ultimately promote three main theses related to *Neurolaw* and its inevitable progress.

### Neuroscience Has Firmly Established Its Place in Jurisprudence

Neuroscience already plays a prominent role in legal proceedings. This trend seems likely to increase rather than decrease; however, this development should not be alarming. As judges and juries are faced with the challenge of weighing this evidence in their decision-making, we have a responsibility to make this process as transparent as possible. This involves promoting better science and better education to judges and legal counsel about these data’s interpretation, limitations, and best practices in quality assurance and analytic strategies.

### Our Normative Understanding of Free Will and Culpability Is Changing

Our understanding of human behavior and free will has steadily incorporated more contributions from science throughout history. The role of neuroscience only represents a recent and specific extension of this progress. Increasingly deterministic models of behavior need not cripple our aim to hold people responsible for their actions, but they arguably drive changes in our normative view of culpability and what constitutes justice.

### Our Legal System Is Evolving, Not Static

Recent changes in our legal system highlight evolving standards in normative judgments regarding the relative value of retributive and rehabilitative interventions. Neuroscience provides a platform to re-assess the value of primarily punitive systems that have historically done little to remedy mental health and social issues that perpetuate high incarceration rates. Rather than eroding jurisprudence, this has the potential to inform more effective policies that serve our society in progressive ways.

## Neuroscience Has Firmly Established Its Place in Jurisprudence

Critical evaluations of the role of neuroscience (and particularly neuroimaging) in legal proceedings abound in both academic publications and the popular media (Brown and Murphy, 2010; Eagleman, 2011a; Morse, 2015; Gonzalez, 2017). While the tone of these pieces can range from cautionary to insolent, they attend to a common issue of what is frequently described as a *meteoric rise* in the consideration of neuroscience-based evidence in courtroom decision-making. They frequently highlight perceived negative consequences of this trend, and some suggest the limited relevance of neuroimaging in court overall. Here we argue that neuroscience and neuroimaging in particular have already established their place in legal proceedings, which is unlikely to subside. A more practical approach for exercising caution in its application will be to improve stakeholders’ understanding of the strengths and limitations of these techniques which includes educating lawyers, judges, and the general public. Educated adoption of neuroscience in legal settings is a practical and realistic solution to any perceived hazards it engenders.

The characterization of a *meteoric rise* of neuroscience used in court carries with it a somewhat menacing connotation that may not be wholly justified. While estimates have suggested an approximate doubling of cases that consider neuroscience data as legal evidence in the past decade (Catley and Claydon, 2016; Farahany, 2016), this is not out of step with its rise in clinical and research settings over the same period (Yeung et al., 2017). Further, in contrast to the tone of many commentaries, this steady increase has not occurred unexpectedly, overwhelming courts with claims that its practitioners cannot fairly evaluate. One of the first considerations of brain imaging as evidence in court occurred over 35 years ago in the high profile trial of John Hinckley Jr. for the attempted assassination of President Ronald Reagan (United States vs. Hinckley, 1982^1^). Brain scans were used in conjunction with other clinical evidence to support Hinckley’s diagnosis of schizophrenia. The brain imaging was not foundational to his diagnosis, but served the purpose of grounding his claims of mental illness in a physical domain (as opposed to purely “psychological”) – an important educational element for jurors in the early 1980s. This context remains among the most influential roles of neuroscience in contemporary jurisprudence, where judges and juries must inevitably weigh the “legitimacy” of health claims and related assertions that remain difficult to account for objectively (e.g., chronic pain, psychiatric disorders). Hinckley was found not guilty by reason of insanity and was committed to a secure hospital for the mentally ill.

Since then, the application of neuroscience evidence in court has increased, but not on a scale that is out of step with advancing scientific knowledge and improved practical utility. Any notions that the rise in neuroscience has happened too quickly for courts to implement its data sagaciously are likely misguided. Several reports have objectively evaluated this changing landscape. Using broad inclusive criteria, it has been estimated that neuroscience evidence is considered in less than 1% of all criminal proceedings (based on court of appeals data) and only about 5% of murder trials dating back to 2005 (Catley and Claydon, 2016; De Kogel and Westgeest, 2016; Farahany, 2016). Contexts that have seen more pronounced increases include evaluation of competency to stand trial, capital cases, and appeals for mitigation of punishment. An observation made frequently across studies is that the rise in neuroscience evidence appears most striking in high-stakes cases. While these numbers are steadily increasing, they do so in-step with advancing scientific understanding and improved potential to inform judges and juries about a number of relevant aspects of mental health, within standard limits of due process – and this context is important. As with any emerging technology, its relevance in legal proceedings must be evaluated carefully with established evidentiary standards (Gaudet, 2011) as courts learn to integrate and adapt to progress in clinical neuroscience. Nonetheless, the utility of neuroscience and neuroimaging data in court is being increasingly realized as counsel, scientists, and practitioners work together to further establish its value in different contexts. Indeed, courts are often recommending, if not requiring, that neuroscience evidence be produced to support arguments being made, when these data are potentially informative (Catley and Claydon, 2016).

Understanding the many possible applications of neuroscience in the courts can help broaden this perspective and allay concerns that its arrival in legal proceedings is premature or imprudent. First, neuroscience evidence in the United States has almost never been an essential factor in determining *actus reus* – whether or not the accused actually committed the criminal act in question. However, for an interesting international example see State of Maharashtra v. Sharma^2^. and commentary by Gaudet (2011). Neuroscience evidence, in the U.S. and worldwide, is far more commonly determined to be relevant for assessing the competency of an individual to stand trial or in addressing the *mens rea* element of criminal liability, but for a more nuanced summary of these various contexts see Slobogin (2017). These latter contexts relating to *mens rea* often require thorough assessments of a defendant’s mental health, which may reasonably include neuroimaging. Defenses built on this reasoning include pleas of insanity, which have evolved substantially over time (see section Our legal system is evolving, not static). Nonetheless, insanity pleas essentially argue that someone was so mentally impaired that s/he was not aware of their own actions, or able to decipher right from wrong. Still, a brain scan may only represent one element of a comprehensive clinical evaluation in such applications, or may be determined to be irrelevant given extant clinical evidence – that is, a brain scan is often not necessary for determining ones mental health status, but may provide supportive evidence. In other cases, neuroimaging may be essential (e.g., brain injury, degenerative disease, tumors). To be sure, insanity pleas overall constitute less than 1% of all felony trials, and their success ordinarily accompanies cases in which a defendant had previously been diagnosed with a mental illness (Kirschner and Galperin, 2001; Perlin, 2016).

As noted above, neuroscience data is also increasingly submitted in the sentencing phase of a trial (e.g., after guilt has already been determined), and evidentiary standards are somewhat more permissive and accommodating during these arguments. This is becoming more common in high stakes sentencing decisions, for instance, in capital cases when the convicted offender faces either the death penalty or life in prison (Miller, 2010). Neuroscience data may then be considered relevant when deciding whether one deserves the harshest possible punishment or a sentence reflecting intervening factors that can include mental health status. Indeed the relevance of mental health in some cases is considered so pertinent that a failure to introduce neuroscience data has been determinative of ineffective counsel and a violation of a defendant’s constitutional right to fair representation (Koenig, 2016).

Additional applications of neuroscience arise in civil cases, which may require demonstrating extent of physical injury. Applications of brain imaging in this context include established documentation of gray and white matter injury with structural brain imaging, but may extend to novel applications that provide information on concussion and mild traumatic brain injury (Vergara et al., 2017), and application of functional imaging techniques addressing chronic pain (Wager et al., 2013). These later examples are emerging areas that need to be vetted further by the scientific community (Davis et al., 2017); however, their appeal and *potential* value is undeniable, underscoring the importance of lawyers and judges remaining in touch with advancing technology. In this way, it is imperative that legal counsel is adequately educated about the relevance and interpretation of neuroscience-based evidence that may aid the fair evaluation of each case.

It should be clear that, in any context, the tools of neuroscience are not subject to more lenient standards than other forms of evidence presented in legal arguments. That is, their probative value must be weighed against the potential for introducing a prejudicial impact or confusion among jurors. Commentaries often use this as a linchpin for their arguments, citing (limited) evidence that brain imaging evidence may mislead jurors and/or distract them from primary lines of reasoning (McCabe and Castel, 2008; Weisberg et al., 2008). Importantly, this evidence has been critically evaluated by others who have noted that these investigations did not present information in a context matching what juries typically encounter (Roskies et al., 2013). Other studies accounting for these factors have indicated no evidence that brain imaging carries any additional weight over and above verbal neuroscience-based testimony (Schweitzer and Saks, 2011; Schweitzer et al., 2011). Further, when evaluated in a legal context where cross-examination critically evaluates the relevance of information, MRI-based evidence is no more persuasive than other (non-neuroscience-based) evidence (McCabe et al., 2011). Finally, the role of the judge as a kind of gatekeeper for admissibility of evidence protects the system from more controversial applications of these tools. This has been demonstrated effectively time and again as courts have rejected the use of fMRI, for example, as a form of lie detection (US v. Semrau, 2010^3^ ; State v Gary Smith 2012^4^.). This has persisted as there is not sufficient scientific consensus for these purposes, at present. As such, the use of fMRI in this context does not pass established Daubert standards of evidence.

The checks and balances built into the U.S. legal system have largely been effective in the face of expanding neuroscience evidence. It should be clear, however, that these safeguards are not intended to unilaterally prevent change in the legal system. Rather, they are intended to promote adaptive interpretation, reflecting normative standards that shift in step with increasing knowledge, advancing technology, and evolving public attitudes (see section “Our legal system is evolving, not static”). As technology continues to improve and new applications arise, it is essential for practitioners of the law to remain adequately informed in order to best serve their roles. The recognition of this imperative is increasingly evident in the resources and attention being devoted to these objectives in recent years (Jones et al., 2013). The MacArthur Foundation Law and Neuroscience project and the Research Network on Law and Neuroscience (MacArthur, 2019) represent large, multimillion dollar investments serving these needs. These efforts accompany many formal educational resources for judges and lawyers that specifically address topics of neuroscience (FJC, 2019), as well as ongoing development of many international conferences and academic societies devoted to increasing scholarship and improving communication within these integrative disciplines.

Our initial assertion in this commentary is intended to be uncontentious. Simply, there are many contexts in which the relevance of neuroscience data is already firmly established, and may in fact be essential for carrying out effective legal decision-making. Most of these applications are not new, but the breadth of their relevance has perhaps widened as their informative value improves in stride with progress in research and the technology itself. The relevance of neuroscience data in jurisprudence shows no evidence of diminishing in the coming years; therefore, we encourage an attitude of integration and motivated legal scholarship. The importance of this is clear even given the limited examples provided here, which leave out additional concerns regarding constitutional principles (Pizzetti, 2011), moral/ethical considerations, e.g., Pallarés-Dominguez and Gonzalez Esteban (2016); Shaw (2018), and Napier (2019), and emerging perspectives in international law (Spranger, 2012). Ongoing critical evaluation of the utility and limits of neuroscience will remain an essential component of this progress. Occasional dismissals of neuroscience’s evolving relevance, in our view, are myopic and potentially dangerous. Criticisms on this order are usually intended to reinforce a static view that the law can continue to operate as effectively without neuroscience, simply because it has in the past. However, this attitude offers little guidance for the inevitable progress facing an assuredly dynamic field, which requires close evaluation of evolving technology and evidence. We therefore reinforce a perspective that the best way for the system to adapt to advancing technology is by improving education and resources available to legal professionals who are increasingly required to incorporate these data in their arguments.

## Our Normative Understanding of Free Will and Culpability Is Changing

Humans have been grappling with the concepts of free will and determinism (or *fatalism*) for most of recorded history (Hoefer, 2016); however, ancient notions of these concepts had little to do with the brain and neuroscience. Instead, philosophers and storytellers alike considered how much of our behavior was controlled by gods, the fates, or other supernatural external forces. Similarly, behavior that was attributable to our own motivations and decisions were not always nested in the brain. Aristotle for instance believed the brain mostly served to cool the blood. Rather, our motivated behavior has historically been attributed to something immaterial like a spirit, soul, or will. As physical sciences improved our understanding of neuromuscular junctions, neurotransmitters, and the role of the brain in organizing behavior based on prior experience, the role of a soul necessarily diminished. In his book *Soul Made Flesh*, Carl Zimmer develops a vivid history of neuroscience around the idea that advances in physiology, medicine, and psychology have incrementally narrowed the role of an immaterial soul as science has increasingly explained biological systems responsible for cognition and behavior (Zimmer, 2005).

In many ways, evolving perspectives about free will represent an extension of this trajectory. As neuroscience offers more detailed and predictive models accounting for human motivations, appetitive drives, and behavioral inhibition, extant descriptions of free will increasingly seem to grasp at something immaterial and elusive. This, somewhat covertly, promotes a paradigm incompatible with natural science, which progresses on a foundation that is fundamentally materialist, reductionist, and determinist in nature. As this represents a predictable extension of prior historical and philosophical progress, the questions neuroscience addresses on this topic are not new ones. However, neuroscience provides an increasingly tangible and convincing platform for demonstrating the limits, proximal antecedents, and illusions that support our subjective sense of free will. The relevance of this for promoting evolving attitudes in jurisprudence relate to how we, as a society, exercise normative judgments about agency, responsibility, and most importantly culpability. Here we illustrate how these attitudes are slowly shifting, and we emphasize the role neuroscience plays in influencing these standards.

Any treatment of how neuroscience has influenced our understanding of free will must address the studies of Benjamin Libet, and perhaps more importantly, contemporary extensions of this work. In the 1980s Libet published research demonstrating the precise timing of one’s subjective perception of making a simple decision to freely move one’s wrist in relation to other physiological events (Libet, 1985). The study essentially recorded three events: the movement of the wrist, the time the participant “decided” to move their wrist, and neural activity surrounding these events. The neural activity indicative of preparations to move one’s wrist was already known (since the 1960s), and is commonly referred to as the readiness potential (Kornhuber and Deecke, 1965). What was striking in Libet’s experiments was the demonstration that this neural preparatory activity consistently preceded one’s subjective sense of having decided to move, by about 350 ms. Preliminary interpretations of these outcomes suggested that neural activity preceding the decision-point constituted evidence of a deterministic process that had already begun, prior to our subjective awareness of it, undermining conventional notions of agency or free will more generally (Libet, 1999). These initial conclusions have been rightfully debated for decades, while others have more quietly continued to improve and expand on these methods.

More recent extensions of this work have included the application of machine learning algorithms to accurately predict subjects’ movements before they decide to move. This has been carried out using intracranial, intracellular recordings within the supplementary motor cortex (Fried et al., 2011). Functional MRI recordings measuring patterns of neural activity across the whole brain have also reliably predicted which of two buttons someone will press up to 7 s before they indicate they’ve decided (Soon et al., 2008). However, other exciting research demonstrates that these behaviors are not determined in such a simplistic way; but rather, they remain influenced by parallel cognitive systems. Executive control systems can essentially veto an intended movement, if given as little as 200 ms warning (Schultze-Kraft et al., 2016). That is, a ‘stop’ signal triggered by a real-time prediction of one’s intended movement is sufficient to allow inhibition and eliminate that movement, provided it is delivered at least 200 ms prior to the execution of the event.

Demonstrations like these provide concrete evidence that our decisions and motivations are accompanied by many parallel neural mechanisms that occupy a dimension beyond our conscious, deliberative processes of reasoning. Measuring the corresponding neural activity provides tangible, proximal measurements of these processes, but neuroscience is not the only context in which we are aware of such unconscious influences on behavior. Freud may have been the first to draw public attention to the prominent role of subconscious influences on our otherwise rational behavior (Freud, 1913). More recently, studies of decision-making in contexts ranging from economics to moral deliberation have made it clear that our choices are strongly guided by implicit emotional influences that often deviate from rational optimization, and the narratives we construct around our decisions are often architected in a *post hoc* manner (Haidt, 2001; Lerner et al., 2015). Finally, we are increasingly aware of the predictable consequences of many remote influences that we have no individual control over. These include our genetic makeup (Brunner et al., 1993; Mason and Frick, 1994), early rearing environments (Kaplow and Widom, 2007; Mulvaney and Mebert, 2007), and complex social systems (Yoshikawa et al., 2012; Javanbakht et al., 2015). These factors all bias our cognition and behavior in predictable ways and their influence impinges on neural systems that guide our behavior directly, in ways that are largely inaccessible to our moment-to-moment conscious, deliberative processes. Better understanding of these influences has, in some ways, also changed the way we reason about culpability.

Challengers to the role of neuroscience in legal contexts will often argue that claims of functional impairments based in neuroscience contribute little to our normative judgments about culpability. This is based, in part, on the rationale that innumerable others with similar impairments have undoubtedly not committed similar crimes, and so the impairment (by itself) is insufficient to predestine the crime (Morse, 2006; Mayberg, 2010). This rebuttal fails to recognize that deterministic influences rarely operate in isolation, and our *normative* judgments ordinarily consider multiple factors and contextual circumstances (Freedman and Zaami, 2019). Further, the deterministic limits of isolated factors on criminal behavior are not uniquely reserved for neuroscientific considerations. This same argument, for instance, fails to undermine the relevance of something like faulty brakes influencing our normative judgments about a fatal car accident, given that faulty brakes only sometimes lead to fatalities, see also (Zeki et al., 2004). This perspective also misses a somewhat more overarching role that neuroscience plays in shifting normative judgments about culpability. That is, neuroscience can help shift our judgments by simply grounding facts about psychological differences in a physical realm, underscoring their contextual relevance among many forms of physical evidence.

If the processes of motivation and decision-making are seen only as imponderable mysteries, inaccessible to reductionistic science, then we are constrained by limited insight into the origins of behaviors we ostensibly wish to diminish in society. We are further bound to make more simplistic normative judgments based on right and wrong, and our interventions will be more unidimensionally focused on reactive punishment. Conversely, integrating deterministic perspectives in explaining behaviors society condemns doesn’t prevent us from using punishment as a deterrent, but only highlights additional points of leverage useful for applying more proactive interventions as an added method of diminishing unwanted behavior, see also Eagleman (2011b) and Slobogin (2011, 2017).

Another interesting context from which to observe this evolving landscape is to consider relatively common forms of pathology that impinge on our ability to choose and behave freely. Fitting examples include obsessive-compulsive disorders and addictions. In both cases, individuals can be said to lose some control over behavior that, in healthy individuals, is attributed to ordinary volitional processes. Normally, washing our hands, going over a mental list, or enjoying a beer are all considered among our ordinary, voluntary, healthy behaviors. Under pathological conditions, however, compulsions to engage in these or other behaviors encroach on (and supersede) other normal motivations. Daily goals, long-term ambitions, and explicit objectives may be at odds with increasingly intrusive thoughts and behaviors that an individual has limited control over. An individual may fully understand, anticipate, and wish to avoid the consequences of certain maladaptive behaviors, while still succumbing to well-worn patterns leading to the undesired behavior. Common understanding about the pathophysiology of these disorders has altered how we address these issues both clinically and interpersonally.

The current accepted model of addiction promoted by the National Institutes of Health is that of a brain disorder instantiated in motivational and inhibitory systems, brought on by exposure to substances that pharmacologically impose lasting physiological changes on these systems (NIDA, 2019). Like other diseases, genetic vulnerabilities, environmental exposures, and variability in physiology all promote individual differences in susceptibility to addiction. Unlike many other diseases, the observable symptoms are almost entirely behavioral. Moreover, these behaviors are often categorically illegal and punishable by law (in the case of illicit drug use), but may also be viewed under a moral lens as a transgression against more virtuous decision making. What makes this acutely relevant to discussions of neurolaw is the nature of arguments for and against the disease model of addiction, and how they reflect philosophical discourse on free will, neuro-determinism, and culpability. Opposition to the disease model can be easily found in publications such as Heyman’s *Addiction: A Disorder of Choice* (Heyman, 2009), Schaler’s *Addiction is a Choice* (Schaler, 2000), and Satel and Lilienfeld’s *Addiction and the Brain-Disease Fallacy* (Satel and Lilienfeld, 2014). These arguments make rhetorical appeals to the primary role of *choice*, *agency*, *volition*, and *self-control*. In doing so, they tacitly place limits on reductionist approaches that examine supportive physiological processes that govern our choices. These arguments seem rooted in the fundamental conservation of free will as something irreducible, and impervious to reductionist, deterministic paradigms.

By contrast, neuroscientific research nested in the disease model of addiction studies elements of motivated behavior in simpler parts, examining individual variability across these dimensions. For example, this research examines shifts in valuation (e.g., the motivational weight of pharmacological reinforcers over natural reinforcers) along with the weakening of inhibitory control (Goldstein and Volkow, 2002). These approaches also examine transitions between behavior governed primarily by executive control systems and behavior carried out by networks governing compulsive, automatic actions (Kalivas and O’Brien, 2008). As such, the “disease” aspect of addiction is more fundamentally rooted in the physiological systems that govern our choices and behaviors, rather than in the complex behavioral symptom of drug-taking *per se*. Adopting this perspective requires a reductionist and determinist paradigm for informing our free will. While not universally accepted (and perhaps still requiring semantic refinement), the progressive contributions of the disease model of addiction include a better understanding of biological influences that culminate in our motivated behavior. Progress on this front further serves to dilute a predominantly moralistic attitude toward addiction that may motivate primarily punitive actions intended to address a very legitimate societal problem. This contribution will feature heavily in our ongoing assessment of the relevance of neuroscience in an evolving criminal justice system.

These arguments are familiar in the context of debates about the nature of free will and responsibility. While our intuitions may still demand the preservation of a concept like free will and agency in our behavior (Nahmias et al., 2005; Nichols, 2011), it has become increasingly necessary to clarify what aspects of our thoughts and behavior remain free, to what extent they are free, and (perhaps most important for our purposes here) what the relevance of this is for our judgments about how to intervene to address pragmatic social needs. After all, moral responsibility is more abstract and partially removed from the practical considerations of punishment and intervention in our justice system. As it turns out, laypeople’s judgments about these topics are not always internally consistent, often reflecting shifting attitudes when considered in abstract terms vs. concrete examples. For instance, when considering theoretical arguments, people are more likely to maintain that determinism undermines basic moral responsibility; when considering concrete episodic scenarios, we are more likely to affirm basic accountability for our actions responsibility (Nichols, 2011).

Using the disease model of addiction as an example, opponents do not deny the evidence of biological changes in motivational systems that account for changes in behavior. However, opponents still cling to the relevance of individual agency, free will, and decision making, ostensibly apart from their biological influences, perhaps only because this reaffirms our most basic intuitions about choice, consequences, and our ability to change (Feldman et al., 2014). This veneration of free will over the biological systems that govern choice may have counterproductive consequences, however. The best methods for intervention arguably improve by understanding the biological systems governing our choices and motivated behavior, particularly in the context of maladaptive behaviors involving substances that impinge directly on these systems. It is the context of intervention that becomes highly relevant for our consideration of the ongoing role of neuroscience in the future of jurisprudence. The influence of neuroscience on these concerns is already evident in a number of contexts discussed in the next section, and it has the potential to continue to improve our practical management of an imperfect but adaptable criminal justice system.

## Our Legal System Is Evolving, Not Static

The relevance neuroscience has in our current justice system is already firmly established in several contexts outlined in Section “Neuroscience Has Firmly Established Its Place in Jurisprudence.” The way neuroscience is promoting progressive changes in our justice system is also evident in a number of ways we address here. We can use recent examples of these changes to help anticipate the ongoing evolution of jurisprudence as informed by advancing neuroscience. Importantly, we reiterate our position that the influence of neuroscience has relatively less to do with any perceived exculpatory extensions of a purely deterministic universe (*my brain made me do it*), and is more practically relevant for the way we interpret concepts like “justice” and the role of the justice system in promoting a safe, functioning society. Shifting normative attitudes on this front influence how we choose to intervene and hold people accountable for their actions. Neuroscience, after all, has improved our general understanding of motivated human behavior and myriad deterministic influences that converge to promote maladaptive, antisocial behavior. Where there is improved understanding of these influences, we will be better equipped to introduce improved strategies at remedying systemic problems contributing to the behaviors and societal problems we aim to diminish.

Conservative appeals to traditional applications of jurisprudence regularly make the claim that neuroscience need not change anything about the way we interpret legal responsibility. This is true in one sense: if our only motivation is preservation of the *status quo*. In an article previously published in this series, *Criminal Responsibility and Neuroscience: No Revolution Yet*, Bigenwald and Chambon (Chambon and Bigenwald, 2019) establish that no revolution is necessary for us to continue applying the same normative framework of responsibility that the legal system has always operated on. Several arguments are presented emphasizing the primacy of our intuitions about agency for assigning criminal responsibility. That is, even the reality of a purely deterministic universe does not negate criminal responsibility, which in their view, exists as a mostly pragmatic concept independent of free will. This is true only in that our legal system certainly employs a number of arbitrary rules in order to remain serviceable. It is no great leap in understanding to suggest that it simply operates ‘as if’ we are responsible agents. Our objection on this matter is that this reality will become increasingly dissatisfying, even from a normative perspective, as general knowledge increases, providing more insight into the boundaries and limitations of our own agency. Fortunately, the present series of articles is under no obligation to preserve the *status quo*; but rather, it challenges us to describe how the legal system might be practically changed by discoveries in neuroscience. We therefore submit that these changes may be less visible in the ways we interpret and enforce the law, and more visible in the ways we punish violators of our laws and adapt as a society to preserve (or advance) our most pressing goals.

As Bigenwald and Chambon point out, *responsibility* has many possible meanings. A tree can certainly be responsible for falling on a wire and causing a power failure, even though it has no real agency. Calling a tree responsible for these consequences doesn’t violate any of our intuitions about agency and its value. Calling a tree “guilty” for this, however, feels odd (violates our intuitions), just as wishing to implement retributive harm on the tree would seem senseless. This illustration emphasizes a division between practical considerations of responsibility and the attribution of a kind of value judgment about the tree’s actions, and how they align with normative moral values. Even as we are keenly interested in (also) preventing other trees from falling (deterrence), one of the key roles of our justice system remains a punitive one, and this features heavily in how harshly we decide to punish. Our intuitions about agency, free will, and moral judgment play a much larger role in our instinct to punish the guilty. Where neuroscience may play its most significant role is in the space between legal determinations and implementing corrective measures that benefit society. Here there remains a great deal of room for improving strategies aimed at protecting and benefiting society on a large scale. These changes in normative attitudes are evident in the evolving standards we use in legal sentencing and the ways we continue to evaluate the relative efficacy of various punitive strategies.

As noted, the criminal justice system in the United States serves many functions beyond a punitive one. We rely on it to deter flagrant abuses of the law, to protect society at large from the most dangerous individuals, and (ostensibly) to help intervene and rehabilitate those who violate the expectations of their social systems. The current implementation of this system, however, has been heavily biased toward retributivist deterrence strategies, which have demonstrated their own limitations over several decades (Frost, 2006). Indeed, they have contributed, in part, to the highest incarceration rates, per capita, in the entire world. Public attitudes play an overt role in this as the Supreme Court has endorsed that public desire for retribution is a legitimate basis for establishing harsh, punitive judgments up to and including capital punishment (Gregg v. Georgia, 1976^5^).

Initial steps in adopting more effective strategies may be fostered by increasing numbers of people reconsidering the implicit relevance and meaning of concepts like *free will* for achieving societies’ goals. As the meaning of this concept evolves and our understanding of behavior integrates more deterministic features, we are less compelled to frame maladaptive, antisocial actions within paradigms that embrace elusive immaterial origins (like evil, for instance) (Grasmick et al., 1992; Unnever et al., 2005). Rather, we are better equipped to recognize the influence of pathology, environment, and acquired maladaptive cognitive strategies in promoting antisocial behavior, where the levers of justice have considerably more remedial influence. After all, pathology is a more tractable problem than is evil. Responding to antisocial behavior, then, becomes a more pragmatic issue, and more progressive strategies aimed at addressing objective moderators of such behavior can be readily explored. In this way, even slow shifts in normative judgments are highly relevant to the way we assess culpability as a society, and the degree to which we view punitive measures as achieving their intended purpose as a remedy against undesired, antisocial behavior.

Neuroscience ultimately provides a useful platform for advocating new strategies of social management, where old strategies have perhaps proven ineffective or inefficient. New strategies may be less oriented toward retribution, *per se*, and more driven by practical concerns serving society with more efficient and productive solutions. Such strategies may, for instance, be aimed at better serving the mental health and social needs of those who come in contact with the justice system, reducing long-term incarceration rates for low risk offenders, and reducing recidivism by improving rehabilitative and reintegration efforts. In the worst scenarios, where rehabilitative interventions may not be a realistic goal, neuroscience also provides a platform for improving our predictions of future dangerousness (Aharoni et al., 2013; Steele et al., 2015; Kiehl et al., 2018). Such strategies may be integrated for making better decisions about those who need to be removed from society permanently. Before addressing this further, it will be important to consider a few examples for how advancing science has already changed the way we think about culpability and make decisions about interventions and punishment as a society.

### Limits on Capital Punishment

Torture and execution have been legally sanctioned forms of punishment since at least the 18th century BCE, as it is indicated in the Code of Hammurabi (c.1750 BCE) for such crimes as burglary, adultery, making false accusations, and poor construction of a house (Harper, 1904). Even within the history of the United States, the use of capital punishment has changed considerably, formerly implemented in cases of burglary, counterfeiting, and treason among others (Randa, 1997). Beginning with the adoption of bans on cruel and unusual punishment^6^., modern societies (including the U.S.) have gradually changed their views on behavior deserving the harshest penalties, limiting its application for the most egregious crimes and even further to individuals most deserving of harsh punishment. Determining who deserves the harshest punishments has a great deal to do with our perceptions of their intentions, malice, and reasonable expectations of self-control. As we will see, these judgments also incorporate the relative utility of the punishment for fulfilling an intended punitive role. The relevance of neuroscience in drawing conclusions about these issues increases as their evaluation increasingly incorporate reductionist, determinist, biological perspectives of motivated behavior.

Prominent examples of this have come in the form of supreme court decisions accompanying restricted applications of the death penalty. For instance, Atkins v. Virginia^7^. ruled to prevent the execution of those with severe intellectual disabilities, citing “evolving standards of decency that mark the progress of a mature society.” In cases like these, these *evolving standards* refer more specifically to what the court witnesses as a consensus among other jurisdictions and the way they have tended to interpret and enforce the law in recent history. Many states, for instance, had previously outlawed the execution of those with severe intellectual disabilities prior to these proceedings. Among the topics discussed in the formal ruling is the sentiment that those with reduced intellectual capacity have limitations in their adaptive functioning, reasoning, communication, and understanding of events around them and the actions of others. Thus, leveraging the most severe of punishments fails to align with the practical concerns of retribution and deterrence.

Similarly, Roper v. Simmons (2005)^8^. abolished capital punishment of juveniles citing similar “evolving standards” and an emerging consensus among other jurisdictions. In this case, however, the decision was also influenced in part by neuroscience research (including fMRI evidence) presented in an amicus brief by the American Psychological Association, suggesting that psychological deficits germane to adolescence (developmental limitations) make young people more prone to impulsive behavior and less capable of the highest order decision-making we ordinarily attribute to adults. That is, opinions informed by progress in neuroscience suggesting a limited capacity for behavioral control are influential for evaluating an individual’s culpability (i.e., how harsh a punishment is justified). Implicit in the developmental perspective applied is an acknowledgment of the capacity for ongoing change. MRI evidence was also presented (in amicus brief) for consideration in Graham v. Florida (2011)^9^. which determined it unconstitutional to sentence juveniles to life without parole for crimes not involving homicide.

These decisions can be fully reconciled with normative attitudes about responsibility and determinism. Despite being fully responsible for their behavior, biological limitations on individuals’ executive functioning and inherent capacity for change play a prominent role in our consideration of how harsh their punishments ought to be. The Court’s decision in Roper v. Simmons affirmed that juveniles have less culpability due to their immature development, making them less deserving of the harshest punishments. These decisions do not imply that, as a society, we are any less interested in protecting ourselves from dangerous people or ensuring the safety of free citizens. What is confirmed in these decisions is a relative diminution in our motivation to levy harsh retributivist judgments in contexts where we recognize deterministic limitations in individual culpability. This, of course, opens the door to consider how we judge those with other biological limitations in cognitive functioning, or those disadvantaged in other ways.

### The Insanity Defense

Our collective understanding of culpability has almost always included provisions for certain disadvantages. A clear illustration of this endures in the limitations on culpability levied against those with serious mental disorders. This has been a common feature of many ancient legal systems and customs, including elements of Roman law which were carried forward in pre-Norman England (Walker, 1985). For instance, it was at times customary for juries to find insane criminals guilty, but refer them to the king for subsequent pardoning. More contemporary applications of these provisions give juries specific guidelines for applying these judgments directly. The *M’Naghten Rule*, for instance, formalized a set of conditions in English law that could be applied more consistently following a controversial acquittal. In 1843, Daniel M’Naghten suffered paranoid delusions and murdered a civil servant, mistaking him for the English Prime Minister. He was acquitted for murder based on substantial evidence that he was mentally ill, and he was forcibly committed to an asylum, where he spent the rest of his life. Despite the very real limits placed on his freedom, the ensuing public dissent following a *not-guilty* verdict (and official condemnation of the verdict by the queen) compelled establishing a set of explicit requirements for instantiating criminal insanity. These guidelines, in some adapted form, are still prevalent in many jurisdictions across the world today. They essentially require (for an insanity defense) that a defendant be so mentally impaired as to not know what they are doing and/or not know right from wrong.

Following in step with the very impetus for M’Naghten, many subsequent adaptations and amendments to these rules have been applied, usually following controversial rulings. As a result, several variations and alternative defenses have been enacted in state and federal jurisdictions. These either amend the essential language of M’Naghten used to describe what constitutes insanity, or they shift the burden of proof in important ways. For instance, in Parsons v. State of Alabama (1887)^10^., an appeal was made following the controversial conviction of Nancy Parsons who killed her husband under the delusion that he had cast an evil spell on her. The court established a provision for instances in which a defendant may be deemed insane, despite knowing right from wrong. The ruling described instances where a disease has “destroyed the defendant’s free will” and became known as the *Irresistible Impulse* defense. Other important developments have included modifications that specifically exclude antisocial personality disorder from an exculpatory mental illness, since its symptoms are primarily manifest through repeated criminal conduct (American Law Institute Model Penal Code, 1962)^11^. There has also been a formal shift of responsibility from the prosecution – proving beyond a reasonable doubt that a defendant was sane – to the defense, which must prove (by preponderance of evidence) that the defendant was insane (Insanity Defense Reform Act; IDRA, 1984)^12^. A lengthy, stand-alone review would be necessary to adequately review the many important modifications that have been made to these rules over time and across many jurisdictions; however, an overarching pattern is apparent in this complex history. Through many shifts of language and interpretations, we continually re-affirm the preservation of limitations on culpability for those impaired by mental illness. We also betray the cognitive dissonance this instills against the backdrop of our most basic retributive motivations, and our sensitivities to potential abuses of these provisions.

As noted above, many of these changes come on the heels of controversial, high-profile cases. Consider for instance the trial of Dan White for the murder of San Francisco Mayor George Moscone and city supervisor Harvey Milk. Despite substantial evidence of premeditation and malice in the killings, White was ultimately convicted of voluntary manslaughter rather than first-degree murder, and served only 5 years in prison. This outcome was aided by what is still disparagingly referred to as “The Twinkie Defense.” To this day, popular retellings of this case often reinforce a narrative that White’s defense asserted his behavior was the result of eating sugary snacks, including Twinkies. In reality, psychiatrists testified that White suffered from major depression and had diminished capacity for controlling his behavior due to this pathology. An incidental detail of his diminished capacity included recent poor dietary habits, despite having been extremely health conscious all his life. The public outcry following his alarmingly lenient sentence was instrumental in abolishing the “diminished capacity” defense in California. The relevance of this trial for our present arguments is not so much to draw attention to the trial and defense, but rather what happened afterward. Following a defense which hinged on severe depression, White served a relatively lenient sentence at Soledad State Prison in California (not a secure hospital, or institute known for therapeutic intervention). Two years after his release from prison, Dan White committed suicide.

These events raise many interesting questions from a legal, psychological, and philosophical perspective. Did White ultimately get what he deserved? Did those seeking harsher retributive action find some gratification in his suicide, or is it inherently less satisfying that White’s death was not carried out in a punitive context? Was White’s case a greater failure in the basic judicial process of determining culpability, or more of a failure in enforcing effective interventions following a determination of his mental illness? Those like White, committing serious offenses (e.g., homicide) in the throes of mental illness, are typically forcibly committed to secure institutions with some focused psychiatric capacity, and do not generally go free in such a short time. From a utilitarian perspective, this form of intervention seems reasonable. It serves the role of protecting society from dangerous people and arguably remains a visible deterrent, while coupling offenders’ containment with therapeutic and/or rehabilitative attention. Where this strategy fails is perhaps only limited in satisfying an instinctual urge to serve harsh retributive actions against those that have harmed us personally and/or violated our most sacred moral values (Grasmick et al., 1992; Unnever et al., 2005).

It matters that this trial has largely been enshrined as a miscarriage of justice, but probably for many of the wrong reasons (as evinced by history’s retelling of the “Twinkie Defense”). In some ways eradication of the diminished capacity defense serves as a scapegoat that only distracts us from more fundamental issues in our society and our justice system that are slow to change. Essentially, we still struggle to balance our shifting attitudes of culpability against a stubborn instinct to enact harsh retributive penalties in cases of egregious tragedy. After all, various provisions for mental illness in sentencing still exist in virtually all jurisdictions. Despite fine tuning the language of these rules, we (as a society) have steadfastly acknowledged that criminal actions occurring due to factors outside the ordinary limits of one’s control deserve more leniency or a categorically different form of intervention than simple retribution. In order to see the potential benefit of progressive changes in jurisprudence, however, our corrections systems and forensic psychiatric facilities need to be equipped with the tools to enact these changes in ways that demonstrate more satisfying results.

### How the Legal System May (Continue to) Change

The contexts described above illustrate that our normative views of culpability have never been static, but continually adapt to evolving standards ushered in by a more refined understanding of human behavior and the boundaries of our own agency. Neuroscience, surely, does not make this progress simpler. On the contrary, as our understanding of biology’s role in promoting pathology and maladaptive behavior increases, this encourages a far more nuanced interpretation of culpability in the face of various advantages and disadvantages. Shifting attitudes on this front have fostered an expansion of contexts that we harbor special provisions for in the law. However, rather than promoting overly exculpatory attitudes (a kind of *straw man* common in arguments diminishing the role of neuroscience in law), these shifts have largely required changes only in the way we intervene and balance corrective and/or punitive measures in such contexts. We suggest it is reasonable to expect these trends to continue as retributive goals are softened and we aim to integrate more practical solutions for addressing criminogenic needs, improving reintegration, and reducing recidivism.

Using offender age as a model for such a transition, the criminal justice system has recently made provisions to limit harsh sentencing (capital punishment/life in prison) of juvenile offenders in most circumstances, but various jurisdictions still apply somewhat arbitrary rules about the cut-offs for these provisions. Certainly young offenders are not all equal from a neurodevelopmental perspective. So does it make sense to apply a bright line rule allowing capital punishment on someone’s 18th birthday? Neuroscience may continue to inform this perspective, expanding a more nuanced evaluation. Recent research, in fact, has demonstrated that a brain-derived measure of gray matter related to age is a better predictor of future antisocial behavior than is chronological age (Kiehl et al., 2018). As such, it may be a more pertinent question to consider the relative advantages in development and mental health with which one is equipped before deciding whether they deserve our harshest punishments. Trends in this direction are encouraged by the bifurcation of guilt and sentencing phases of some criminal trials. Another perspective to consider is what effort and resources are justified in the aim of preserving and enacting capital punishment as an extreme punitive measure for rare circumstances. Studies on the deterrence effects of the death penalty are equivocal at best (Weisberg, 2005), and economic scrutiny suggests that we may be incapable of enacting the death penalty in any reasonably efficient manner such that it serves its intended purposes (Aviram and Newby, 2013). Despite our enduring retributivist instincts, we may eventually decide that abolition of the death penalty represents a more practical solution, obviating some of our more difficult choices when it comes to punishment. But capital punishment is not the only context within which shifting attitudes may promote more pragmatic strategies.

In the case of less severe sentences, we (as a society) have demonstrated more amenability to the potential value of remedial approaches and possible re-integration of young offenders. Certainly, more aggressive treatment strategies integrating contemporary cognitive-behavioral approaches for improving long-range outcomes have proven both successful and cost-effective (Caldwell and Van Rybroek, 2013). Consider for instance progressive treatment programs being instituted at the Mendota Juvenile Treatment Center among high-risk young offenders (Caldwell and Van Rybroek, 2005; Caldwell et al., 2006a). Analyses have indicated better outcomes and relatively less economic burden on society by enacting these aggressive treatment strategies (Caldwell et al., 2006b). To be sure, there will always be those who are resistant to our best available treatments at any given time, and unable to return safely to free society. However, this further reinforces the value of pursuing new and better strategies informed by ongoing research addressing the origins, development, and maintenance of maladaptive, antisocial behavior.

In assessing how neuroscience may continue to inform judicial decision-making in the future, many possibilities arise. Could brain scans that objectively quantify one’s neurodevelopment or functional capacity eventually be used to determine whether one is tried as an adult or juvenile? Could predictive models determine whether one is amenable to therapeutic attention or is likely to remain resistant to available rehabilitative efforts? Could neuroscientific measures reveal specific risk factors for re-offending that are not evident on standard psychiatric assessments? These are difficult questions indeed, and ones that we do not yet have answers for. We simply argue that to ignore them or to undermine their potential value only to reinforce the *status quo* seems myopic and overtly servile toward an imperfect system. As neuroscience ushers in a more complete bio-psycho-social understanding of maladaptive behavior, and as ongoing incarceration strategies become unsustainable, our prediction is that we will be forced to consider alternative approaches that serve public interest in more pragmatic ways. This will involve wider application of therapeutic, rehabilitative approaches and more aggressive therapeutic and reintegration strategies that reduce the likelihood for recidivism. Such applications may be particularly effective among young offenders (Glenn, 2019). This may also include better risk assessment in making decisions about sentencing and parole. Major advances on these fronts may only require us to first suspend our most basic retributivist instincts when addressing social problems, and remain open minded about the potential for more prudent strategies. Neuroscience doesn’t fill these roles on its own, but it provides a platform for advancing each of these goals through empirical research and improved knowledge.

## Author Contributions

NA developed the primary theses and arguments presented in this review. KK provided additional insights, commentary, and editorial remarks.

## Conflict of Interest

The authors declare that the research was conducted in the absence of any commercial or financial relationships that could be construed as a potential conflict of interest.

## Footnotes

1 *United States v. Hinckley*, 525 F. Supp. 1342 (D.C. 1981).

2 *State of Maharashtra v. Sharma*, C.C., No. 508/07, Pune, June 12, 2008 (India).

3 *US v. Semrau*, 693 F.3d 510 (6th Cir. 2012).

4 *Smith v. State*, 32 A.3d 59, 423 Md. 573 (2011) (pretrial testimony).

5 *Gregg v. Georgia*, 428 U.S. 153, 96 S. Ct. 2909, 49 L. Ed. 2d 859 (1976).

6 U.S. Const. amend. VIII.

7 *Atkins v. Virginia*, 536 U.S. 304, 122 S. Ct. 2242, 153 L. Ed. 2d 335 (2002).

8 *Roper v. Simmons*, 543 U.S. 551, 125 S. Ct. 1183, 161 L. Ed. 2d 1 (2005).

9 *Graham v. Florida*, 560 U.S. 48, 130 S. Ct. 2011, 176 L. Ed. 2d 825 (2010).

10 *Parson v. State*, 81 Ala. 577, 2 So. 854, 2 So. 2d 854 (1887).

11 American Law Institute: Model Penal Code. Philadelphia: ALI, (1962) Ref. 11, § 4.01

12 Insanity Defense Reform Act (“IDRA”), 18 U.S.C. § 17(b) (1984).

## References

[B1] Aharoni E. Vincent G. M. Harenski C. L. Calhoun V. D. Sinnott-Armstrong W. Gazzaniga M. S. (2013). Neuroprediction of future rearrest. *Proc. Natl. Acad. Sci. U.S.A.* 110 6223–6228. 10.1073/pnas.1219302110 23536303 PMC3625297

[B2] Aviram H. Newby R. (2013). Death row economics: the rise of fiscally prudent anti-death penalty activism. *Crim. Just.* 28 33–40.

[B3] Brown T. Murphy E. (2010). Through a scanner darkly. *Stanford Law Rev.* 62 1119–1208. 20429137

[B4] Brunner H. G. Nelen M. Breakefield X. Ropers H. Van Oost B. (1993). Abnormal behavior associated with a point mutation in the structural gene for monoamine oxidase A. *Science* 262 578–580. 10.1126/science.8211186 8211186

[B5] Burns K. Bechara A. (2007). Decision making and free will: a neuroscience perspective. *Behav. Sci. Law* 25 263–280. 10.1708/2631.27049 17393405

[B6] Caldwell M. Skeem J. Salekin R. Van Rybroek G. (2006a). Treatment response of adolescent offenders with psychopathy features: a 2-year follow-up. *Crim. Justice Behav.* 33 571–596. 10.1177/0093854806288176

[B7] Caldwell M. F. Van Rybroek G. (2013). Effective treatment programs for violent adolescents: programmatic challenges and promising features. *Aggress. Violent Behav.* 18 571–578. 10.1016/j.avb.2013.06.004

[B8] Caldwell M. F. Van Rybroek G. J. (2005). Reducing violence in serious juvenile offenders using intensive treatment. *Int. J. Law Psychiatry* 28 622–636. 10.1016/j.ijlp.2004.07.001 16112731

[B9] Caldwell M. F. Vitacco M. Van Rybroek G. J. (2006b). Are violent delinquents worth treating? A cost–benefit analysis. *J. Res. Crime Delinq.* 43 148–168. 10.1177/0022427805280053

[B10] Catley P. Claydon L. (2016). The use of neuroscientific evidence in the courtroom by those accused of criminal offenses in England and Wales. *J. Law Biosci.* 2 510–549. 27774211 10.1093/jlb/lsv025 PMC5034405

[B11] Cave S. (2016). *There’s no Such Thing as Free Will: But We’re Better Off Believing in it Anyway. The Atlantic.* Available online at: www.theatlantic.com/magazine/archive/2016/06/theresno-such-thing-as-free-will/480750/?utm_source=atlfb (accessed November 15, 2019).

[B12] Chambon V. Bigenwald A. (2019). Criminal responsibility and neuroscience: no revolution yet. *Front. Psychol.* 10:1406. 10.3389/fpsyg.2019.01406 31316418 PMC6610327

[B13] Davis K. D. Flor H. Greely H. T. Iannetti G. D. Mackey S. Ploner M. (2017). Brain imaging tests for chronic pain: medical, legal and ethical issues and recommendations. *Nat. Rev. Neurol.* 13 624–638. 10.1038/nrneurol.2017.122 28884750

[B14] De Kogel C. Westgeest E. (2016). Neuroscientific and behavioral genetic information in criminal cases in the Netherlands. *J. Law Biosci.* 2 580–605. 27774213 10.1093/jlb/lsv024 PMC5034396

[B15] Eagleman D. (2011a). *The Brain on Trial. The Atlantic.* Available online at: https://www.theatlantic.com/magazine/archive/2011/07/the-brain-on-trial/308520/ (accessed November 15, 2019).

[B16] Eagleman D. (2011b). The brain on trial. *Atlantic* 7 112–123.

[B17] Farahany N. A. (2016). Neuroscience and behavioral genetics in US criminal law: an empirical analysis. *J. Law Biosci.* 2 485–509. 27774210 10.1093/jlb/lsv059 PMC5034387

[B18] Feldman G. Baumeister R. F. Wong K. F. E. (2014). Free will is about choosing: the link between choice and the belief in free will. *J. Exp. Soc. Psychol.* 55 239–245. 10.1016/j.jesp.2014.07.012

[B19] FJC (2019). *Educational Resources, Neuroscience* [Online]. Washington, DC: Federal Judicial Center.

[B20] Freedman D. Zaami S. (2019). Neuroscience and mental state issues in forensic assessment. *Int. J. Law Psychiatry* 65:101437. 10.1016/j.ijlp.2019.03.006 30952490

[B21] Freud S. (1913). *The Interpretation of Dreams.* New York, NY: The Macmillan Company.

[B22] Fried I. Mukamel R. Kreiman G. (2011). Internally generated preactivation of single neurons in human medial frontal cortex predicts volition. *Neuron* 69 548–562. 10.1016/j.neuron.2010.11.045 21315264 PMC3052770

[B23] Frost N. A. (2006). *Punitive State: Crime, Punishment, and Imprisonment Across the United States.* El Paso, TX: LFB Scholarly Publishing.

[B24] Gaudet L. M. (2011). Brain fingerprinting, scientific evidence, and Daubert: a cautionary lesson from India. *Jurimetrics* 51 293–318.

[B25] Glenn A. L. (2019). Using biological factors to individualize interventions for youth with conduct problems: current state and ethical issues. *Int. J. Law Psychiatry* 65:101348. 10.1016/j.ijlp.2018.04.008 29673560

[B26] Goldstein R. Z. Volkow N. D. (2002). Drug addiction and its underlying neurobiological basis: neuroimaging evidence for the involvement of the frontal cortex. *Am. J. Psychiatry* 159 1642–1652. 10.1176/appi.ajp.159.10.1642 12359667 PMC1201373

[B27] Gonzalez R. (2017). *How Criminal Courts are Putting Brains–Not People–on Trial. Wired.* Available online at: https://www.wired.com/story/how-criminal-courts-are-putting-brains-not-people-on-trial/ (accessed November 15, 2019).

[B28] Grasmick H. G. Davenport E. Chamlin M. B. Bursik R. J. Jr. (1992). Protestant fundamentalism and the retributive doctrine of punishment. *Criminology* 30 21–46. 10.1111/j.1745-9125.1992.tb01092.x

[B29] Haidt J. (2001). The emotional dog and its rational tail: a social intuitionist approach to moral judgment. *Psychol. Rev.* 108 814–834. 10.1037/0033-295x.108.4.814 11699120

[B30] Harper R. F. (1904). *The Code of Hammurabi King of Babylon.* Chicago, IL: The University of Chicago Press.

[B31] Harris S. (2012). *Free Will.* New York, NY: The New York Times.

[B32] Heyman G. M. (2009). *Addiction: A disorder of Choice.* Cambridge, MA: Harvard University Press.

[B33] Hoefer C. (2016). “Causal Determinism,” in *The Stanford Encyclopedia of Philosophy*, ed. Zalta E. N. (Stanford, CA: Stanford University).

[B34] Javanbakht A. King A. P. Evans G. W. Swain J. E. Angstadt M. Phan K. L. (2015). Childhood poverty predicts adult amygdala and frontal activity and connectivity in response to emotional faces. *Front. Behav. Neurosci.* 9:154. 10.3389/fnbeh.2015.00154 26124712 PMC4464202

[B35] Jones O. D. Marois R. Farah M. J. Greely H. T. (2013). Law and neuroscience. *J. Neurosci.* 33 17624–17630. 24198354 10.1523/JNEUROSCI.3254-13.2013 PMC6618431

[B36] Kalivas P. W. O’Brien C. (2008). Drug addiction as a pathology of staged neuroplasticity. *Neuropsychopharmacology* 33 166–180. 10.1038/sj.npp.1301564 17805308

[B37] Kaplow J. B. Widom C. S. (2007). Age of onset of child maltreatment predicts long-term mental health outcomes. *J. Abnorm. Psychol.* 116 176–187. 10.1037/0021-843x.116.1.176 17324028

[B38] Kiehl K. A. Anderson N. E. Aharoni E. Maurer J. M. Harenski K. A. Rao V. (2018). Age of gray matters: neuroprediction of recidivism. *Neuroimage Clin.* 19 813–823. 10.1016/j.nicl.2018.05.036 30013925 PMC6024200

[B39] Kirschner S. M. Galperin G. J. (2001). Psychiatric defenses in New York county: pleas and results. *J. Am. Acad. Psychiatry Law* 29 194–201. 11471786

[B40] Koenig E. G. (2016). A fair trial: when the constitution requires attorneys to investigate their clients’ Brains. *Fordham Urban Law J.* 31 177–225.

[B41] Kornhuber H. H. Deecke L. (1965). Hirnpotentialänderungen bei willkürbewegungen und passiven bewegungen des menschen: bereitschaftspotential und reafferente potentiale. *Pflüger’s Archiv Gesamte Physiologie Menschen Tiere* 284 1–17. 10.1007/bf00412364 14341490

[B42] Lerner J. S. Li Y. Valdesolo P. Kassam K. S. (2015). Emotion and decision making. *Annu. Rev. Psychol.* 66 799–823. 25251484 10.1146/annurev-psych-010213-115043

[B43] Libet B. (1985). Unconscious cerebral initiative and the role of conscious will in voluntary action. *Behav. Brain Sci.* 8 529–539. 10.1017/s0140525x00044903

[B44] Libet B. (1999). Do we have free will? *J. Conscious. Stud.* 6 47–57.

[B45] MacArthur (2019). *Research Network on Law and Neuroscience* [Online]. Available online at: http://www.lawneuro.org/ (accessed December 2, 2019).

[B46] Mason D. A. Frick P. J. (1994). The heritability of antisocial behavior: a meta-analysis of twin and adoption studies. *J. Psychopathol. Behav. Assess.* 16 301–323. 10.1007/bf02239409

[B47] Mayberg H. (2010). “Does neuroscience give us new insights into criminal responsibility,” in *A Judge’s Guide to Neuroscience: A concise Introduction*, ed. Gazzaniga M. (Santa Barbara, CA: University of California), 37–41.

[B48] McCabe D. P. Castel A. D. (2008). Seeing is believing: the effect of brain images on judgments of scientific reasoning. *Cognition* 107 343–352. 10.1016/j.cognition.2007.07.017 17803985

[B49] McCabe D. P. Castel A. D. Rhodes M. G. (2011). The influence of fMRI lie detection evidence on juror decision−making. *Behav. Sci. Law* 29 566–577. 10.1002/bsl.993 21751243

[B50] Miller G. (2010). Brain exam may have swayed jury in sentencing convicted murderer. *Science.* Available online at: https://www.sciencemag.org/news/2010/12/brain-exam-may-have-swayed-jury-sentencing-convicted-murderer (accessed November 15, 2019).

[B51] Morse S. J. (2006). Brain overclaim syndrome and criminal responsibility: a diagnostic note. *Ohio State J. Crim. Law* 3 397–412.

[B52] Morse S. J. (2015). “Neuroscience, free will, and criminal responsibility,” in *Faculty Scholarship Paper 1604*, ed. Glannon W. (Philadelphia, PA: University of Pennsylvania Law School: Penn Law: Legal Scholarship Repository).

[B53] Mulvaney M. K. Mebert C. J. (2007). Parental corporal punishment predicts behavior problems in early childhood. *J. Fam. Psychol.* 21 389–397. 10.1037/0893-3200.21.3.389 17874924

[B54] Nahmias E. Morris S. Nadelhoffer T. Turner J. (2005). Surveying freedom: folk intuitions about free will and moral responsibility. *Philos. Psychol.* 18 561–584. 10.1080/09515080500264180

[B55] Napier S. (2019). The minimally conscious state, the disability bias, and the moral authority of advance directives. *Int. J. Law Psychiatry* 65:101333. 10.1016/j.ijlp.2018.03.001 29661479

[B56] Nichols S. (2011). Experimental philosophy and the problem of free will. *Science* 331 1401–1403. 10.1126/science.1192931 21415346

[B57] NIDA (2019). *The Science of Drug Use and Addiction: The Basics* [Online]. North Bethesda, MD: The National Institute on Drug Abuse.

[B58] Pallarés-Dominguez D. Gonzalez Esteban E. (2016). The ethical implications of considering neurolaw as a new power. *Ethics Behav.* 26 252–266. 10.1080/10508422.2015.1012763

[B59] Pardo M. S. Patterson D. (2010). Philosophical foundations of law and neuroscience. *Univ. Ill. Law Rev.* 2010 1211–1250.

[B60] Perlin M. L. (2016). “The insanity defense: Nine myths that will not go away,” in *The Insanity Defense: Multidisciplinary Views on its History, Trends and Controversies*, ed. White M. D. (New York, NY: New York Law School).

[B61] Pizzetti F. G. (2011). In quest of constitutional principles of “Neurolaw”. *Medicina Secoli* 23 963–990. 23057208

[B62] Randa L. E. (1997). *Society’s Final Solution: A History and Discussion of the Death Penalty.* Lanham, MD: University Press of America.

[B63] Roskies A. L. Schweitzer N. J. Saks M. J. (2013). Neuroimages in court: less biasing than feared. *Trends Cogn. Sci.* 17 99–101. 10.1016/j.tics.2013.01.008 23428934

[B64] Sapolsky R. M. (2017). *Behave: The Biology of Humans at Our Best and Worst*, Chapter 16 New York, NY: Penguin Random House, 580–613.

[B65] Satel S. Lilienfeld S. O. (2014). Addiction and the brain-disease fallacy. *Front. Psychiatry* 4:141. 10.3389/fpsyt.2013.00141 24624096 PMC3939769

[B66] Schaler J. A. (2000). *Addiction is a Choice.* Peru, IL: Carus by Open Court.

[B67] Schultze-Kraft M. Birman D. Rusconi M. Allefeld C. Görgen K. Dähne S. (2016). The point of no return in vetoing self-initiated movements. *Proc. Natl. Acad. Sci. U.S.A.* 113 1080–1085. 10.1073/pnas.1513569112 26668390 PMC4743787

[B68] Schweitzer N. J. Saks M. J. (2011). Neuroimage evidence and the insanity defense. *Behav. Sci. Law* 29 592–607. 10.1002/bsl.995 21744379

[B69] Schweitzer N. J. Saks M. J. Murphy E. R. Roskies A. L. Sinnott-Armstrong W. Gaudet L. M. (2011). Neuroimages as evidence in a mens rea defense: no impact. *Psychol. Public Policy Law* 17 357–393. 10.1037/a0023581

[B70] Shaw E. (2018). Counterproductive criminal rehabilitation: dealing with the double-edged sword of moral bioenhancement via cognitive enhancement. *Int. J. Law Psychiatry* 65:101378. 10.1016/j.ijlp.2018.07.006 30206004

[B71] Slobogin C. (2011). Prevention as the primary goal of sentencing: the modern case for interdeterminate dispositions in criminal cases. *San Diego L. Rev.* 48:1127.

[B72] Slobogin C. (2017). Neuroscience nuance: dissecting the relevance of neuroscience in adjudicating criminal culpability. *J. Law Biosci.* 4 577–593. 10.1093/jlb/lsx033 29868186 PMC5965501

[B73] Soon C. S. Brass M. Heinze H.-J. Haynes J.-D. (2008). Unconscious determinants of free decisions in the human brain. *Nat. Neurosci.* 11 543–545. 10.1038/nn.2112 18408715

[B74] Spranger T. E. (2012). *International Neurolaw: A Comparative Analysis.* Heidelberg: Springer-Verlag.

[B75] Steele V. R. Claus E. D. Aharoni E. Vincent G. M. Calhoun V. D. Kiehl K. A. (2015). Multimodal imaging measures predict rearrest. *Front. Hum. Neurosci.* 9:425. 10.3389/Fnhum.2015.00425 26283947 PMC4522570

[B76] Unnever J. D. Cullen F. T. Applegate B. K. (2005). Turning the other cheek: reassessing the impact of religion on punitive ideology. *Justice Q.* 22 304–339. 10.1080/07418820500089091

[B77] Vergara V. M. Mayer A. R. Damaraju E. Kiehl K. A. Calhoun V. (2017). Detection of mild traumatic brain injury by machine learning classification using resting state functional network connectivity and fractional anisotropy. *J. Neurotrauma* 34 1045–1053. 10.1089/neu.2016.4526 27676221 PMC5333571

[B78] Vincent N. A. (2013). *Neuroscience and Legal Responsibility.* New York, NY: Oxford University Press.

[B79] Wager T. D. Atlas L. Y. Lindquist M. A. Roy M. Woo C.-W. Kross E. (2013). An fMRI-based neurologic signature of physical pain. *N. Engl. J. Med.* 368 1388–1397. 10.1056/NEJMoa1204471 23574118 PMC3691100

[B80] Walker N. (1985). The insanity defense before 1800. *Ann. Am. Acad. Polit. Soc. Sci.* 477 25–30. 10.1177/0002716285477001003 11616555

[B81] Weisberg D. S. Keil F. C. Goodstein J. Rawson E. Gray J. R. (2008). The seductive allure of neuroscience explanations. *J. Cogn. Neurosci.* 20 470–477. 10.1162/jocn.2008.20040 18004955 PMC2778755

[B82] Weisberg R. (2005). The death penalty meets social science: deterrence and jury behavior under new scrutiny. *Annu. Rev. Law Soc. Sci.* 1 151–170. 10.1146/annurev.lawsocsci.1.051804.082336

[B83] Yeung W. Goto T. K. Leung W. K. (2017). A bibliometric review of research trends in neuroimaging. *Curr. Sci.* 112:725 10.18520/cs/v112/i04/725-734

[B84] Yoshikawa H. Aber J. L. Beardslee W. R. (2012). The effects of poverty on the mental, emotional, and behavioral health of children and youth: implications for prevention. *Am. Psychol.* 67 272–284. 10.1037/a0028015 22583341

[B85] Zeki S. Goodenough O. Sapolsky R. M. (2004). The frontal cortex and the criminal justice system. *Philos. Trans. R. Soc. Lond. B Biol. Sci.* 359 1787–1796. 15590619 10.1098/rstb.2004.1547 PMC1693445

[B86] Zimmer C. (2005). *Soul Made Flesh: The Discovery of the Brain–and How it Changed the World.* New York, NY: Simon and Schuster.

